# Sclerostin and Cardiovascular Disease

**DOI:** 10.1007/s11914-023-00810-w

**Published:** 2023-07-25

**Authors:** Jonathan H. Tobias

**Affiliations:** 1 Musculoskeletal Research Unit, Translational Health Sciences, Bristol Medical School, University of Bristol, Bristol, UK; 2 MRC Integrative Epidemiology Unit, Population Health Sciences, Bristol Medical School, University of Bristol, Bristol, UK

**Keywords:** Atherogenesis, Wnt signalling, Romosozumab, Randomised controlled trial, Mendelian randomisation

## Abstract

**Purpose of Review:**

The role of wnt signalling in atherogenesis raises the possibility that the wnt inhibitor, sclerostin, provides a natural defence to this process, and that anti-sclerostin antibodies might increase the risk of atherosclerosis and associated conditions such as CVD. This article aims to triangulate evidence concerning possible adverse effects of sclerostin inhibition on CVD risk.

**Recent Findings:**

Randomised controlled trials of treatment with the anti-sclerostin antibody, romosozumab, have yielded conflicting evidence with respect to possible adverse effects of sclerostin inhibition on CVD risk. To further examine the causal relationship between sclerostin inhibition and CVD risk, three Mendelian randomisation (MR) studies have examined effects of sclerostin lowering on CVD outcomes, using common genetic variants in the *SOST* gene which produces sclerostin, to mimic effects of a randomised trial. Concordant findings were seen in two studies, comprising an effect of sclerostin lowering on increased risk of MI and type II diabetes mellitus. One study also suggested that sclerostin lowering increases coronary artery calcification.

**Summary:**

Triangulation of evidence from different sources provides some suggestion that sclerostin lowering increases MI risk, supporting the need for CVD risk assessment when considering treatment with romosozumab.

## Introduction

The discovery that sclerostin deficiency underlies the rare bone disorder sclerosteosis was the first indication that this protein plays a key role in regulating bone mass [1, 2]. Subsequent studies revealed that sclerostin, of which osteocytes are the primary source, plays an important role in mechanosensory responses of the skeleton; deformation due to mechanical loading reduces sclerostin secretion, leading to increased bone formation as a result of reduced inhibition of wnt pathways involved in osteoblastogenesis [3]. Reduced sclerostin-mediated inhibition of wnt pathways also acts to suppress bone resorption. Abrogation of sclerostin activity was subsequently found to increase bone mass in a variety of animal models. This led to the development of monoclonal anti-sclerostin antibodies for treating osteoporosis, of which romosozumab has received marketing approval. This followed two pivotal phase three trials, both of which demonstrated impressive gains in bone mineral density (BMD) and reductions in fracture risk in postmenopausal women receiving romosozumab [4, 5••]. However, adverse event recording in one of these studies revealed an excess risk of myocardial infarction (MI) and stroke [5••] in the romosozumab group. This finding led to concerns that as well as improving bone mass, sclerostin inhibition increases the risk of cardiovascular disease (CVD), reflecting a previously unsuspected role of sclerostin in cardiovascular tissue. The present paper aims to triangulate evidence concerning the risk of CVD following sclerostin inhibition, based on a narrative review of experimental studies, observational investigations, clinical trials, and Mendelian Randomisation (MR) studies.

## Experimental Studies

Though predominantly expressed in bone, sclerostin is also expressed in vascular tissue, including at sites of vascular calcification [6, 7]. The wnt pathway plays an important role in the trans-differentiation of vascular smooth muscle cell towards an osteoblastic/calcifying phenotype [8]. Since sclerostin acts as a wnt inhibitor, taken together, these observations raise the possibility that sclerostin plays a protective role in atherogenesis. In line with this suggestion, upregulation of sclerostin was found to inhibit the development of atherosclerosis in a mouse model [9]. Moreover, *SOST* knockout mice were found to develop more extensive vascular calcification compared to wild-type mice, following administration of an adenine-containing diet to promote chronic kidney disease [10•]. Whereas sclerostin was observed to be expressed at sites of vascular calcification in atherogenic mouse models, it is unclear whether this is a mediatory or reactive process [11]. In experimental studies intended to evaluate potential CVD toxicity of romosozumab specifically, no adverse effects were observed on vascular calcification or any related CVD outcome in cynomolgus monkeys or rats, or in mouse models of accelerated atherosclerosis [12]. Hence, whereas sclerostin may have a protective role against atherogenesis, there may be some redundancy given the lack of adverse effect of sclerostin inhibition with romosozumab in experimental studies.

## Observational Studies

### Summary of Findings

Several previous studies have examined associations between circulating levels of sclerostin and CVD, vascular calcification, and CVD risk factors. A recent meta-analysis revealed no overall association between circulating sclerostin levels and cardiovascular events [13]. On the other hand, in a study not included in this meta-analysis, higher sclerostin levels were found to be positively associated with coronary artery disease severity, and risk of subsequent cardiac death, in 2000 patients from Germany (mean age 63) undergoing coronary angiography [14]. Consistent with the latter finding, a recent meta-analysis reported a positive association between serum sclerostin levels and risk of coronary artery and/or abdominal aortic calcification [13]. In terms of relationships between circulating levels of sclerostin and CVD risk factors, in a meta-analysis combining the mixed German angiography cohort described above, with a younger population-based female only UK cohort (mean age 48), higher levels of sclerostin were found to be associated with greater risk of diabetes mellitus, higher triglycerides, poorer renal function and lower HDL levels [14].

### Interpretation of Findings

Taken together, observational studies suggest that if anything, sclerostin levels are positively related to CVD and related risk factors. However, rather than implying that sclerostin inhibition is likely to have a beneficial (as opposed to negative) influence on these outcomes, the direction of associations seen observationally may not necessarily reflect causal effects. This is exemplified by relationships between sclerostin and BMD, since whereas sclerostin levels are considerably elevated in those with high bone mass [15], sclerostin acts causally to lower BMD [16]. The positive association between sclerostin and BMD in observational studies might also contribute to the positive observational association between sclerostin levels and CVD risk through confounding. Such an explanation would require that BMD is also positively associated with CVD. However, osteoporosis is generally considered to be positively related to CVD risk, implying an inverse relationship with BMD [17]. That higher BMD per se does not predict greater CVD risk is an important assumption for MR studies described below, where in one such study genetic instruments for sclerostin exposure were selected based on their relationship with BMD [18].

## Clinical Trials

### Summary of Findings

In the ARCH trial, postmenopausal women were randomised to romosozumab or alendronate for 12 months, followed by treatment with alendronate in both groups for a further 12 months [5••]. Those randomised to romosozumab had an increased odds (1.31) of reporting a major adverse CVD event (MACE). This included an odds ratio of 2.65 (95%CI 1.03, 6.77) for cardiac ischaemic events and 2.27 (0.93, 5.22) for cerebrovascular events. A signal for increased cardiovascular risk was also observed in a placebo-controlled trial of romosozumab for 12 months in men, with MACE rates twofold higher in the active treatment arm (4.9% versus 2.5%) [19]. A caveat to both these studies is that the studies were not powered to look at rare adverse events and since absolute CVD event rates were very low, it is always possible that differences between groups may have been due to chance events. In contrast, MACE events were balanced in the other pivotal phase three trial of romosozumab in postmenopausal women, FRAME, which in contrast to ARCH used a placebo as the comparator [4]. Moreover, a recent meta-analysis of four and six studies on MACE (relative risk 1.14; 0.83, 1.57) and CVD-related death (relative risk 0.92; 0.53, 1.59) respectively found no effect of romosozumab in postmenopausal women [13].

### Interpretation of Findings

The CVD event data in the ARCH trial was examined in detail by the European Medicines Agency [20]. Over the initial 12 months of the study, MACE event rates in the alendronate group were if anything lower than expected given the age of participants and their relatively high prevalence of CVD risk factors. One possible explanation is that rather than an adverse effect of romosozumab, those randomised to alendronate benefited from a protective effect of bisphosphonates. Previous findings from a randomised controlled trial of zoledronate in older participants, which revealed a reduction in all-cause mortality, raised the possibility that bisphosphonates may as a class protect against CVD [21]. However a subsequent meta-analysis of bisphosphonates was not suggestive of any such protective effect [22].

## Postmarketing and Surveillance Data

Japan has been a useful source of observational and surveillance data, following the launch of romosozumab in March 2019. A recently published observational study of Japanese patients with total exposure to romosozumab of 39,352 person-years found no increased risk of stroke or CVD [23]. On the other hand, postmarketing surveillance data has identified a number of predominantly male cases where initiation of romosozumab was linked to MI, stroke or cardiac death [24, 25•]. The majority of these events occurred in Japan, where prior cardiovascular event is not a contra-indication to commencing romosozumab.

## Mendelian Randomisation Studies

### Summary of Findings

Patients with sclerosteosis often die at a relatively young age due to complications of raised intracranial pressure as a consequence of overgrowth of the skull bones; however, there are no reports of effects on CVD risk either in this condition or the closely related Van Buchem’s disease which is also associated with sclerostin deficiency [26]. However, the small number of patients with these conditions makes it difficult to draw firm conclusions. Three studies have used genetics to infer relationships between sclerostin and CVD risk, using an MR framework based on common single nucleotide polymorphisms (SNPs) [18, 27, 28•] (see Table 1). All three investigations examined the effects of common SNPs within the *SOST* gene responsible for producing sclerostin (i.e. *cis* SNPs). In addition, these studies attempted to model the effect of administration of a sclerostin inhibitor such as romosozumab, by examining the effect of lower sclerostin on cardiovascular outcomes. Findings differed in that two studies pointed to an increased risk of MI and related risk factors with lower sclerostin [18, 28•], whereas Holdsworth et al. only found evidence of an increase in systolic blood pressure [27].

**Table 1:** Findings from Mendelian randomisation studies of effects of sclerostin lowering on cardiovascular risk

Outcome	Bovijn [18]* Odds ratio	*P*	Holdsworth [27]** Odds ratio	*P*	Zheng [28•]*** Odds ratio	*P*
Coronary artery disease	1.10 (1.00–1.20)	0.04	0.999 (0.990, 1.007)	0.727	1.01 (0.74, 1.39)	0.954
Myocardial infarction	1.18 (1.06–1.32)	0.003	1.003 (0.987, 1.018)	0.731	1.35 (1.01, 1.79)	0.04
MACE	1.12 (1.03–1.21)	0.0007	N/A		N/A	
Ischaemic stroke	N/A		1.003 (0.991, 1.016)	0.604	0.95 (0.48, 1.87)	0.874
T2DM	1.15 (1.05–1.27)	0.003	1.009 (1.000, 1.019)	0.061	1.32 (1.03, 1.69)	0.030
Hypertension	1.12 (1.05–1.20)	8.9 x 10^−4^	0.998 (0.990, 1.006)	0.653	1.02 (0.98, 1.06)	0.311
Outcome	Beta (95% CI; clinical units)	*P*	Beta (SD)	*P*	Beta (SD)	*P*
Coronary artery calcification	N/A		N/A		0.236 (0.110)	0.033
Systolic blood pressure	1.33 (0.76–1.91) mmHg	5.9 x 10^−6^	0.0040 (0.0006, 0.0075)	0.022	N/A	
Diastolic blood pressure	− 0.01 (− 0.37, 0.35) mmHg	0.95	-0.0026 (-0.0062, 0.0010)	0.162	N/A	
Triglycerides	9.58 (1.33–17.84) mg/dl	0.02	0.0011 (− 0.0024, 0.0047)	0.542	0.107 (0.082)	0.190
LDL cholesterol	− 1.11 (− 4.91,2.69) mg/dl	0.57	0.0021 (− 0.0015, 0.0057)	0.263	-0.008 (0.044)	0.857
HDL cholesterol	− 1.37 (− 2.88, 0.13) mg/dl	0.07	− 0.0013 (− 0.0047, 0.0022)	0.480	− 0.092 (0.052)	0.073

*MACE* major adverse cardiovascular events includes MI and/or coronary artery revascularization, stroke and death from either

*Estimates scaled to effect of romosozumab 210 monthly for 12 months on LS BMD in ARCH and FRAME trials

**Estimates per cardiac/arterial SOST expression lowering allele

***Estimates per SD decrease in sclerostin

Bovijn et al. [18] found that sclerostin lowering increased the risk of MI, MACE, type 2 diabetes mellitus (T2DM) and hypertension, increased triglyceride levels and reduced HDL (Table 1). Their genetic instrument for sclerostin lowering was based on two conditionally independent *SOST* SNPs identified from a genome-wide association study (GWAS) of BMD estimated from heel ultrasound (eBMD) in UK Biobank [29], and subsequently confirmed to be associated with lumbar spine BMD in a further GWAS [30]. A genetic instrument using these SNPs was scaled based on the lumbar spine BMD gain following romosozumab 210 monthly in a phase II trial in Japanese postmenopausal women [31].

Zheng et al. reported similar findings [28•]. The genetic instrument in this study was based on five independent *SOST* SNPs identified in a GWAS of circulating sclerostin in 34,000 Europeans, scaled to a one SD decrease in circulating sclerostin levels [28•]. A more recently available GWAS of prior MI was used based on UK Biobank (*n* approx. 472,000) [32]. In addition, Zheng et al. had access to results from a large GWAS of coronary artery calcification (CAC) [33], which provided further evidence of an effect of sclerostin lowering on CVD. A further instrument was also evaluated combining the most strongly associated *SOST* SNP with three *trans* SNPs (i.e. SNPs located in genes other than *SOST*) identified from the sclerostin GWAS, including the *B4GALNT3* locus, the strongest genetic signal for circulating sclerostin. These latter analyses identified an effect of sclerostin inhibition on risk of hypertension.

Holdsworth et al found no effect of sclerostin lowering on risk of MI or T2DM, or lipid levels, and just found an effect on systolic blood pressure [27]. The genetic instrument in this study comprised three *SOST* SNPs associated with both reduced *SOST* mRNA expression in arterial tissue, and higher eBMD in a more recent GWAS [34]. Associations with cardiovascular outcomes were subsequently examined per *SOST* expression lowering allele [27].

### Interpretation of Findings: Sclerostin Inhibition and Risk of CVD

Whether associations between the *SOST* SNPs selected by Bovijn et al. and CVD and related risk factors might result from linkage with neighbouring genes has been questioned [35, 36]. That said, despite the different methods used in these three studies for selection of *SOST* genetic instruments, associated with distinct assumptions and limitations, there was considerable correlation between the *SOST* SNPs used in these three studies, and indeed Holdsworth et al and Zheng et al had one *SOST* SNP in common [28•]. This makes it somewhat difficult to explain why the studies by Bovijn and Zheng et al. had concordant results, but differed from those of Holdsworth et al. Whereas Bovijn et al. and Zheng et al. used scaling strategies as described above, no scaling strategy was employed by Holdsworth et al. Since effect sizes of common variants are relatively small, the absence of any scaling methods by Holdsworth et al. would be expected to result in relatively small effect sizes; however, the level of statistical evidence supporting any given effect, as reflected by *P* values, would be unaffected.

Intriguingly, the point estimate of the effect of sclerostin lowering on MI risk observed by Zheng et al. (odds ratio 1.35) was very similar to that observed for MACE events in the ARCH trial (relative risk 1.31) [5••]. Conceivably, the effect of an SD decrease in circulating sclerostin as estimated by Zheng et al could have been broadly equivalent to an effect of romosozumab 210 mg/month administration in ARCH. On the other hand, MR analyses examine the effect of lifelong exposure to lower sclerostin, in contrast to clinical use where sclerostin inhibitors are administered for just 12 months. To the extent that sclerostin inhibition increases cardiovascular risk, the signal observed from MR studies to date suggests this may be restricted to cardiac events as opposed to stroke, though this may be more a reflection of methodological limitations in evaluating the latter. For example, in the study by Zheng et al., only two *SOST* SNPs were shared with the stroke outcome GWAS datasets.

### Interpretation of Findings: Sclerostin Inhibition and Atherogenic Risk Factors

The finding from Zheng et al. that sclerostin lowering increases CAC is consistent with the suggestion above that sclerostin directly inhibits vascular calcification. On the other hand, observations that sclerostin lowering increases the risk of T2DM suggests that indirect mechanisms may also be involved. How sclerostin inhibition might influence risk of T2DM is unclear. The wnt pathway, which sclerostin inhibits, is also known to have a role in regulating adipogenesis [37], which is likely to impact on insulin sensitivity and hence risk of T2DM. Consistent with this suggestion, though Bovijn et al. found no effect of sclerostin inhibition on BMI, a positive effect was observed on waist/hip ratio adjusted for BMI [18]. Additionally, as well as effects on bone formation, sclerostin is thought to stimulate bone resorption via a RANKL-dependent pathway [38], which is predicted to release undercarboxylated osteocalcin from the bone matrix. Undercarboxylated osteocalcin has been shown to stimulate pancreatic insulin secretion resulting in improved glucose homeostasis and reduced T2DM risk [39–41].

### Interpretation of Findings: Cis Versus Trans Effects

**Fig. 1:**
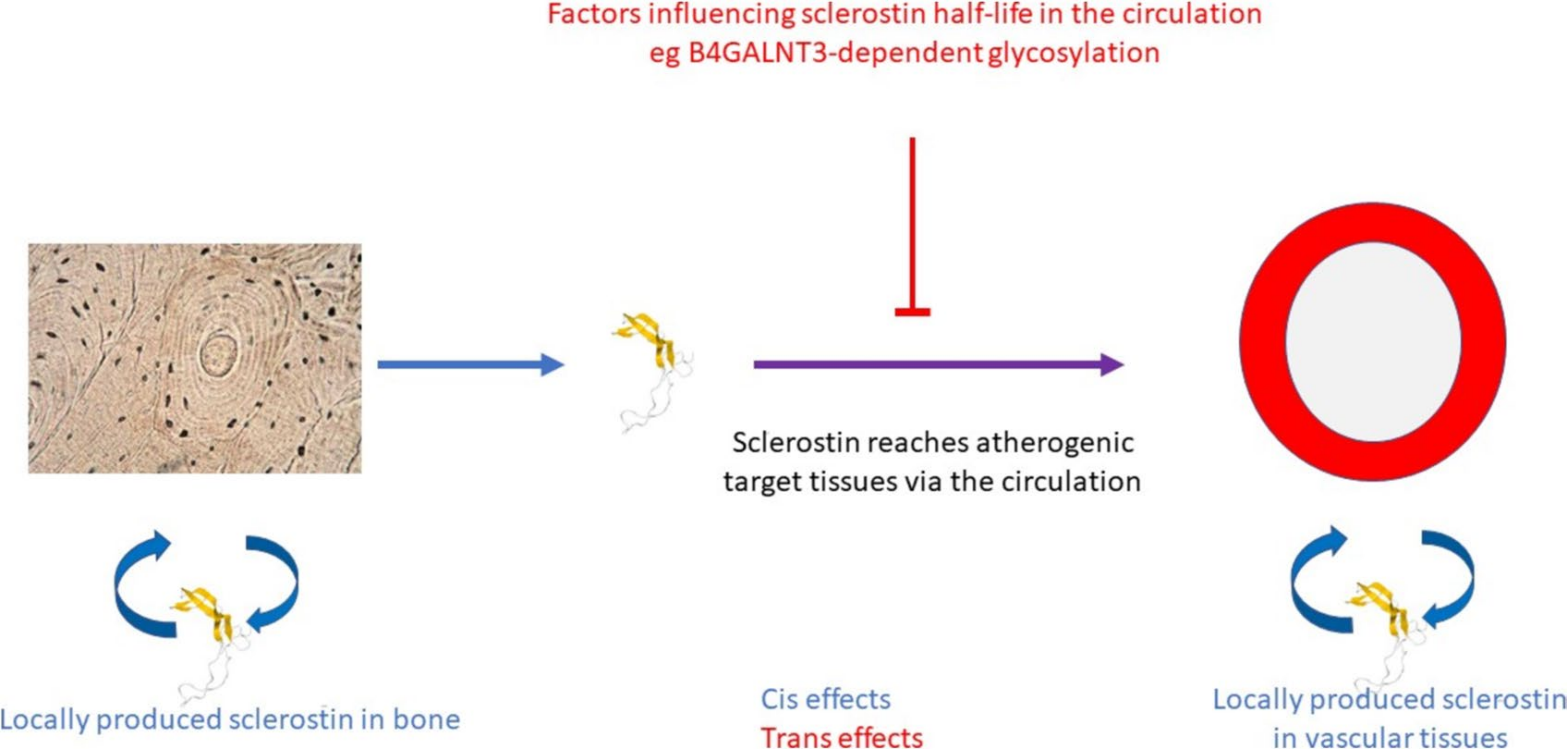
Proposed genetic influences on circulating sclerostin levels acting via *cis* mechanisms (i.e. by influencing expression of the *SOST* gene responsible for producing sclerostin) or *trans* mechanisms (i.e. by acting via other genes). The latter are exemplified by the enzyme B4GALNT3 which is thought to influence sclerostin clearance from the circulation as a consequence of effects on N-terminal glycosylation

Circulating sclerostin levels, as instrumented in the study by Zheng et al. [28•], reflect the net effect of sclerostin production and clearance. Presumably, *cis* SNPs within the *SOST* gene influence sclerostin production in tissues via effects on transcriptional efficiency and mRNA expression. In terms of *trans* SNPs, *B4GALNT3* was the strongest genetic signal for circulating sclerostin, and contributed to the relationship between with sclerostin levels, eBMD and fracture risk [16]. Precisely how the protein product beta-1,4-N-acetylgalactosaminyltransferase 3 (B4GALNT3), a glycosylation enzyme, affects circulating sclerostin levels is unclear. B4GALNT3 transfers N-acetylgalactosamine (GalNAc) onto N-acetylglucosaminebeta-benzyl to form GalNAcβ1,4-GlcNAc structures on protein epitopes (LDN-glycosylation). Sclerostin undergoes B4GALNT3-dependent LDN-glycosylation, and *B4GALNT3* null mice have substantially increased circulating sclerostin levels as well as reduced bone mass and bone strength [42]. These effects could conceivably involve altered production or clearance of sclerostin, reflecting glycosylation-dependent cellular export and systemic degradation respectively, both of which are well recognised. That a *B4GALNT3* instrument had no detectable effect on CAC or CVD risk, in contrast with a *SOST* instrument, might reflect the fact that this action is mediated by reduced local production (*cis* effect) as opposed to increased plasma clearance (*trans* effect) (see Fig. 1).

## Strategies for Mitigating Against Increased CVD Risk Following Sclerostin Inhibition

Taken together, the evidence discussed above provides some concern that sclerostin inhibition with romosozumab may increase the risk of CVD events and associated risk factors. To an extent, this is already mitigated against, since marketing authorisation for romosozumab in the USA and Europe states previous MI or stroke as a contra-indication. However, an important question is whether patients who are at increased risk of MI or stroke, but who are yet to sustain such an event, should also be counselled against taking romosozumab. National UK guidance suggests that rather than an absolute contra-indication, a small excess in cardiovascular risk, for example arising from smoking, should be included as part of a broader conversation when weighing up risks and benefits of treatment [43]. Cardiovascular risk calculators such as Qrisk3 and the European Society of Cardiology Heartscore offer a useful means for individualised cardiovascular risk calculation in this context. The latter may be particularly helpful as it identifies a high risk category of individuals which could be used to define patients in whom romosozumab should be avoided.

## Summary

Findings that sclerostin is expressed in arterial tissue and acts as a wnt inhibitor, and that the wnt pathway plays a role in the trans-differentiation of vascular smooth muscle cell towards an osteoblastic/calcifying phenotype, provides circumstantial evidence that sclerostin produced in arterial tissue may provide a natural defence mechanism against atherosclerosis. Therefore, there is a reasonable basis for considering that pharmacological inhibition of sclerostin carries a potential risk of accelerating atherosclerosis, leading to an increased risk of CVD. Given these theoretical concerns, clinical data related to the anti-sclerostin antibody, romosozumab has been carefully scrutinised. An excess in MACE events was observed in those receiving romosozumab in a large trial in postmenopausal women, and a smaller trial in men, using alendronate and placebo as the comparator respectively. In contrast, several other clinical studies showed no elevation in CVD events, including a large observational study. However, postmarketing surveillance from Japan, where past MI or stroke is not a contra-indication, has identified a number of instances where initiation of romosozumab has been linked to MI or stroke, particularly in men. Two out of three MR studies provided further evidence for such a risk, with those genetically predisposed to lower sclerostin found to be at increased risk of MI and T2DM, and one study finding greater CAC.

## Conclusions

Triangulation of evidence from laboratory, clinical and genetic studies provides some suggestion that sclerostin inhibition is associated with an increased risk of CVD, though findings are not entirely consistent. Hence, it would seem prudent to apply strategies to mitigate against increased CVD risk when prescribing romosozumab. Whereas marketing authorisation already states past MI and stroke as contra-indications in many countries, other indicators of CVD risk may also need to be taken into account.
